# Observing elusive tetroxides in gas-phase radical reactions supports the Russell mechanism

**DOI:** 10.1126/sciadv.aeb6495

**Published:** 2026-03-13

**Authors:** Barbara Nozière, Roger Patrick

**Affiliations:** ^1^ Royal Institute of Technology (KTH), Department of Chemistry, Stockholm, Sweden.; ^2^ Chemical Kinetics Research, PO Box 390103, Mountain View, CA 94039, USA.

## Abstract

Organic peroxy radicals (RO_2_) are important intermediates for oxidation processes in aerobic chemical systems. Their self- and “cross”-reactions (i.e., with themselves and other RO_2_) are increasingly receiving attention in a wide range of applications, from atmospheric chemistry to cancer therapies. However, their mechanism has been debated for decades. The Russell mechanism, widely assumed for these reactions today, is characterized by a tetroxide intermediate, only observed once and partially since its postulate in 1957. Here we report the observation of tetroxides in the gas-phase reactions of different RO_2_ by direct mass spectrometry, in which ionic and gas-phase dimerization could be ruled out. Within the uncertainties in the kinetic profiles, the lifetime for CH_3_OOOOCH_3_ was determined to be in the range 0.2 and 200 milliseconds, consistent with an intermediate and supporting the Russell mechanism.

## Teaser

Organic tetroxides were observed in gas-phase reactions of RO_2_ radicals, supporting the Russell mechanism postulated in 1957.

## INTRODUCTION

**Fig. 1.**
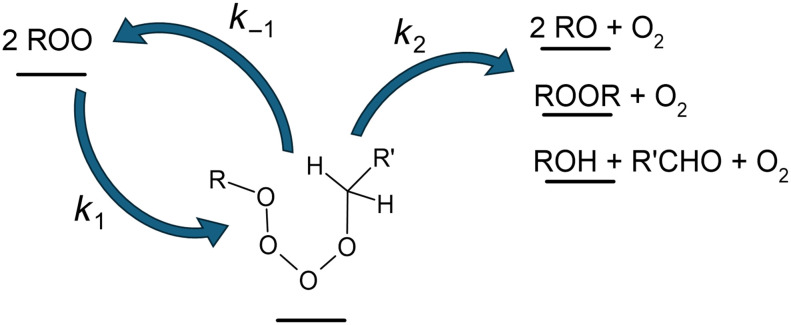
Russell mechanism. Schematics of the Russell mechanism for the self-reaction of organic peroxy radicals, RO_2_, showing the cyclic tetroxide intermediate and its main reactions.

Organic peroxy radicals (RO_2_) are key intermediates in the oxidation of organic compounds in a wide range of aerobic chemical systems, including living organisms and oxidative stress (*1*, *2*), low temperature combustion (*3*), and Earth’s atmosphere (*4*, *5*). While in these systems, their self- and cross-reactions are not usually their main fate, they can play important roles. For instance, they are increasingly being considered in cancer therapy for their in situ production of excited oxygen, O_2_ (^1^Δ) (*6*). In Earth’s atmosphere, they are suspected to form low-vapor pressure products, leading to the nucleation of aerosol particles of climate importance (*7*). However, many aspects of these reactions remain unclear today, limiting the knowledge of their contribution to these chemical systems and the development of their applications. Open questions include the wide variation of the RO_2_ reactivity with their structure (*8*), the singlet or triplet state of the oxygen molecule produced (*9*, *10*), and the potential existence of a channel producing diperoxides, ROOR (*11*, *12*). These questions reflect the limited knowledge of the reaction mechanism. The one widely admitted today is the Russell mechanism, which was hypothesized in 1957 (*13*) and is characterized by the formation of a cyclic tetroxide intermediate, ROOOOR (Fig. 1)

$${\text{RO}}_{2}+{\text{RO}}_{2}\to \text{ROOOOR}$$ (Rxn. 1)

$$\text{ROOOOR}\to {\text{RO}}_{2}+{\text{RO}}_{2}$$ (Rxn. −1)

ROOOOR→reaction products (Rxn. 2)

After being rejected for about 30 years (*8*), this mechanism is now supported by a number of theoretical studies (*8*, *11*, *12*, *14*–*17*). However, its experimental validation is, by and large, based on observing stable products (*9*, *18*–*22*). To our knowledge, the observation of the tetroxide intermediate was only reported once and only tentatively (*23*): In a matrix-isolation study of CH_3_O_2_, infrared lines were attributed to the vibrations expected for its tetroxide, CH_3_OOOOCH_3_, assuming a linear structure. However, the identity of this compound was not confirmed. Most experimental studies of the Russell mechanism were also performed in condensed phase and at low temperature and concluded that tetroxide intermediates are only stable at *T* < 193 K (*18*, *20*).

In this work, we report the observation of self- and cross-tetroxides (R_2_O_4_ and RR′O_4_) in the gas-phase reactions of a range of RO_2_ at room temperature (*T* = 300 ± 4 K) using real-time proton transfer mass spectrometric technique previously optimized to detect RO_2_ (*24*, *25*). The identity of these compounds was confirmed by series of tests, including isotopically labeled radicals and ruling out the potential contribution of ionic or gas-phase dimerization to the observations. To obtain further information on these tetroxides, the formation and decomposition kinetics for CH_3_OOOOCH_3_ was also studied and the results compared to theoretical values using quantum calculations.

## RESULTS

### Observing and identifying tetroxides in the gas-phase reactions of RO_2_

#### Optimizing tetroxide detection with proton transfer mass spectrometry

**Fig. 2.**
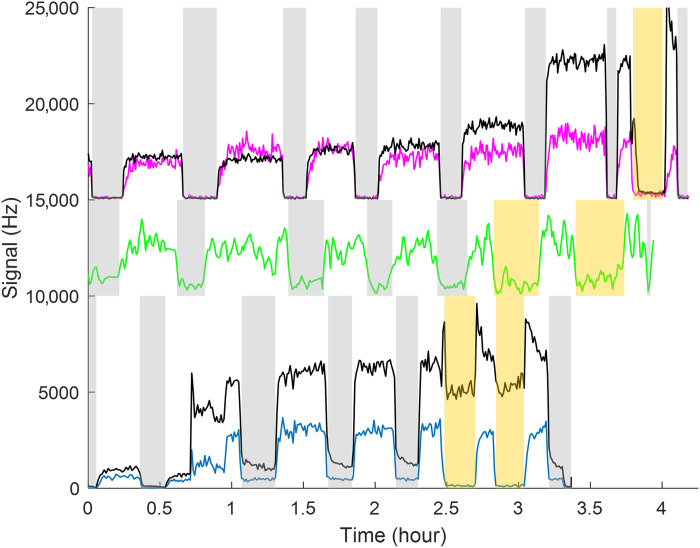
Time profiles for the tetroxides (R_2_O_4_) in the self-reactions of CH_3_O_2_, C_2_H_5_O_2_, and their cross-reaction. (**Top**) Self-reaction of CH_3_O_2_. Black: CH_3_O_2_ (×0.45), pink: C_2_H_6_O_4_ (×2). (**Middle**) Green: C_4_H_10_O_4_ (×20) in the self-reaction of C_2_H_5_O_2_. (**Bottom**) Cross-reaction between C_2_H_5_O_2_ and CH_3_O_2_. Black: CH_3_O_2_ (×0.25), blue: C_2_H_5_O_4_CH_3_ (×0.5). In all cases, the tetroxide signal follows that of the RO_2_: It disappears when the lights are OFF (no RO_2_ produced, gray areas) and when NO is added to the reactor because the reaction RO_2_ + NO consumes the RO_2_ and takes over the RO_2_ self- and cross-reactions (no tetroxides produced. Orange areas). Examples of profiles for all the tetroxides and RO_2_ studied in this work are presented in fig. S2.

The formation of self- and cross-tetroxides was investigated in this work in the gas-phase reactions of CH_3_O_2_, ^13^CH_3_O_2_, CD_3_O_2_, C_2_H_5_O_2_, and iso-C_3_H_7_O_2_, in flow reactors in the laboratory (Materials and Methods and table S1). The compounds present in the outflow of the reactor were observed with proton transfer mass spectrometry (ion list in table S2). This technique was shown previously to be efficient in detecting alkyl peroxy radicals in the gas (*24*, *25*). More recently, operating with milder ionization conditions, such as a high drift tube pressure (15 to 20 Torr) and low electrical energy (20 to 30 Td), was shown to favor the detection of organic peroxides (ROOR) (*26*). Studying the reaction of CH_3_O_2_ with the same ionization conditions also led to the observation of ions corresponding to the formula C_2_H_6_O_4_(H_2_O)H^+^ and C_2_H_6_O_4_(H_2_O)_2_H^+^ [mass/charge ratio (*m/z*) 113 and 131, respectively, table S2) thus to the tetroxide CH_3_OOOOCH_3_. A higher drift tube pressure and low electrical energy was also shown to favor the detection of these ions (fig. S1). Using these conditions to study the self- and cross-reactions of other RO_2_ systematically led to the observation of ions corresponding to the general formula R_2_O_4_(H_2_O)H^+^ [or RR′O_4_(H_2_O)H^+^] in all the experiments (Fig. 2, fig. S2, and table S2).

#### Attributing the observed ions to R_2_O_4_ compounds

The first part of this work consisted in demonstrating that the observed R_2_O_4_(H_2_O)H^+^ and RR′O_4_(H_2_O)H^+^ ions resulted from actual compounds present in the reaction mixtures and not from secondary ionization processes. This was established on the basis of three main arguments presented below.

The first one was that secondary ionization is energetically unfavorable for RO_2_ which, combined with their small concentration, made it negligible in this work. This was based on the fact that, in the experiments (fig. S3), very few ions were observed that could be attributed to ion dimers, except potentially for the most intense reaction product in each reaction (CH_3_OH with CH_3_O_2_ and carbonyl compound with all the other RO_2_). However, no ion dimer could be observed for the other stable products (fig. S3), implying that ion dimerization should be negligible for the RO_2_ since they were in even smaller concentration. An even stronger argument ruling out ion dimerization for RO_2_ was obtained by comparing the thermodynamics of their ion dimerization with that of reference compounds: acetone, acetonitrile, and acetaldehyde. For this, the primary and secondary ionization processes for a compound A were considered. In our experiments, the primary ionization process is the proton transfer with (H_2_O)_n_H^+^, with *n* = 1 to 3

$$A+{\left(H_{2}O\right)}_{n}H^{+}⇄A{\left(H_{2}O\right)}_{n-m}H^{+}+m\left(H_{2}O\right),\text{with}\;m\leq n$$ (Rxn. 3)

The secondary ionization process potentially accounting for the observed R_2_O_4_(H_2_O)H^+^ ions is the ion dimerization

$$A+A{\left(H_{2}O\right)}_{n-m}H^{+}⇄A_{2}{\left(H_{2}O\right)}_{n-m-p}H^{+}+p\;\left(H_{2}O\right)$$ (Rxn. 4)

Both Rxns. 3 and 4 are fast and lead to an equilibrium between A, its monomer ion A(H_2_O)_n-m_H^+^, and its dimer ion, A_2_(H_2_O)_n-m-p_H^+^. Combining the expressions for the equilibrium constants *K*_3_ and *K*_4_ for each reaction gives

$$\begin{array}{c} \left[A_{2}{\left(H_{2}O\right)}_{n-m-p}H^{+}\right]=(K_{4}/K_{3})\times \\ {\left[A{\left(H_{2}O\right)}_{n-m}H^{+}\right]}^{2}\times {\left[H_{2}O\right]}^{m-p/}\;\left[{\left(H_{2}O\right)}_{n}H^{+}\right] \end{array}$$ (Eq. 1)

The concentrations [(H_2_O)_n_H^+^] and [H_2_O] being constant throughout the experiments for given ionization conditions, Eq. 1 shows that the concentration of dimer ion A_2_(H_2_O)_n-m-p_H^+^ increases as the square of that of the monomer ion A(H_2_O)_n-m_H^+^. The signal for each ion being proportional to its concentration, *S*_dimer_ ∝ [A_2_(H_2_O)_n-m-p_H^+^] and *S*_monomer_ ∝ [A(H_2_O)_n-m_H^+^], Eq. 1 implies that

$$S_{\text{dimer}}/{S^{2}}_{\text{monomer}}\propto K_{4}/K_{3}$$ (Eq. 2)

The ratio of the equilibrium constants *K_4_/K_3_* is specific to compound A and contains the thermodynamic information describing its efficiency in producing ion dimers. It can be expressed as

$$K_{4}/K_{3}=B\;e^{-\left(\Delta \text{H4}-\Delta \text{H3}\right)/\mathrm{RT}}$$ (Eq. 3)

where Δ*H_3_* and Δ*H_4_* are the Δ*H* for Rxns. 3 and 4 respectively, and *B* a constant. ln(*K*_4_*/K*_3_) should thus be proportional to *(*Δ*H_4_ −* Δ*H_3_*).

Varying the concentration of acetone, acetonitrile, and acetaldehyde under the same ionization conditions than in the RO_2_ experiments confirmed the apparition of ion dimers at large concentration [>parts per million (ppm)], with *S*_dimer_ increasing as the square of *S*_mononer_, as expected from Eq. 2 (fig. S4). Reporting the values of ln(*S*_dimer_*/S^2^*_monomer_) obtained with each compound against Δ*H*_3_ (kilojoules per mole) calculated from their respective proton affinity (*27*) showed an excellent correlation (fig. S5). This indicated that Δ*H*_4_ was either much smaller than Δ*H*_3_ or scaled up with it and that the different detection sensitivities for the different compounds and ions had a negligible impact compared to the large changes in Δ*H* between compounds. Extrapolating this trend to the RO_2_, for which Δ*H*_3_ ~ −25 kJ mol^−1^ (*28*) gave a ratio *S*_dimer_/*S*^2^_monomer_ ~ 9.6 × 10^−8^ Hz^−1^, much smaller than those for acetone, acetaldehyde, and acetonitrile (fig. S5). This indicated that ion dimerization was energetically much less favorable for RO_2_ than for these stable compounds. Applying this ratio of *S*_dimer_/*S*^2^_monomer_ to the RO_2_ signals in the experiments predicted negligible signals for their dimer ion. For instance, in the self-reaction of CH_3_O_2_ (fig. S3), *S*_monomer_ = *S*_CH3O2_ = 3400 Hz; thus, *S*_dimer_ = *S*_C2H6O4_ = 9.6 × 10^−8^ × (3400)^2^ = 1.1 Hz. By comparison, the observed R_2_O_4_(H_2_O)H^+^ signal was about 200 Hz. Ion dimerization was thus concluded to be negligible in our experiments and not to account for the observed R_2_O_4_(H_2_O)H^+^ signals. Note that this argument does not apply to iso-C_3_H_7_O_2_ because its concentration was in the ppm range and its R_2_O_4_ signals were very small. However, ion dimerization was ruled out for this radical based on the other arguments presented below.

The second argument supporting that the R_2_O_4_(H_2_O)H^+^ ions resulted from actual compounds was their water cluster distribution. Under the ionization conditions of this study, proton transfer resulted exclusively in ion water clusters M + 19 [“A(H_2_O)H^+^”] and M + 37 [“A(H_2_O)_2_H^+^”] but never in M + 1 ions (table S2), while ion dimerization resulted exclusively M + 1 ions (i.e., “AH^+^” ions) (fig. S4). The “R_2_O_4_” ions observed in the experiments could only be attributed a formula R_2_O_4_(H_2_O)H^+^ (i.e., M + 19 water clusters) because a formula (R_2_H_2_O_5_)H^+^ (i.e., M + 1 ions) could not be accounted for by combining two RO_2_ molecules or even one RO_2_ and a stable reaction product. Furthermore, for the reaction of CH_3_O_2_, isotopic labeling unambiguously established that these ions had the formula C_2_H_6_O_4_(H_2_O)H^+^ and not (C_2_H_8_O_5_)H^+^ (cf. the next section). The fact that the observed R_2_O_4_ ions had an M + 19 formula strongly supported that they resulted from actual compounds present in the reactor rather than from secondary ionization processes.

The third argument ruling out ion dimerization as a source for the R_2_O_4_(H_2_O)H^+^ ions was their lack of systematic correlation with the RO_2_ signals in the experiments (fig. S6). Because ion-neutral reactions such as Rxn. 4 are fast, secondary ions are usually strongly correlated with their parent ions, as confirmed for acetone, acetonitrile, and acetaldehyde (fig. S4). By contrast, in 20 of 28 experiments (70%), the correlation coefficient is less than 0.5 (fig. S6), ruling out ion dimerization as a source for R_2_O_4_(H_2_O)H^+^. The R_2_O_4_(H_2_O)H^+^ ions observed in the experiments were thus concluded to result from actual compounds of formula R_2_O_4_ present in the reaction mixtures.

#### Identifying the R_2_O_4_ compounds as tetroxide intermediates

Once it was established that the R_2_O_4_(H_2_O)H^+^ ions resulted from actual compounds of formula R_2_O_4_, further tests and experiments were performed to identify these compounds. First, the possibility that they could be weakly bound gas-phase dimers of RO_2_ (“RO_2_.RO_2_”) was examined. The equilibrium constant between RO_2_ and their gas-phase dimers, *K*_RO2.RO2_, which is not known, was estimated from that of HO_2_, *K*_HO2-HO2_ (300 K) = 2.5 × 10^−16^ cm^−3^ (*29*) and noting that it is ~500 times larger than its complexation constant with water, *K*_HO2-H2O_ (300 K) = 5.2 × 10^−19^ cm^−3^ (*30*). Using the complexation constant with water for CH_3_O_2_ of *K*_RO2.H2O_ = 1.54 × 10^−21^ cm^−3^ (*31*) thus gave *K*_RO2.RO2_ ~8 × 10^−19^ cm^−3^ = [RO_2_.RO_2_] / [RO_2_]^2^. Thus, a typical concentration in the experiments of [RO_2_] = 10^12^ molec. cm^−3^ gives [RO_2_.RO_2_] ~8 × 10^5^ molec. cm^−3^, thus a negligible concentration of gas-phase dimers for RO_2_ and not accounting for the observed R_2_O_4_ compounds.

The R_2_O_4_ compounds were specifically identified as products of the reactions of RO_2_ by observing that they disappeared when the photolysis lights were turned off (no RO_2_ produced, gray areas in Fig. 2 and fig. S2) and when NO was added in excess in the reactor and the reaction RO_2_ + NO replaced RO_2_ + RO_2_ (orange areas in Fig. 2 and fig. S2). The formation of cross-tetroxides of formula RR′O_4_ in the presence of two different RO_2_ further confirmed that these compounds were specifically produced by the self- and cross-reactions of the RO_2_. All these observations evidenced the occurrence of a general mechanism systematically producing R_2_O_4_ (or RR′O_4_) compounds in the self- and cross-reaction of all RO_2_.

Investigating the self-reaction of isotopes of CH_3_O_2_, ^13^CH_3_O_2_, and CD_3_O_2_ showed the formation of R_2_O_4_ compounds with two (*m*/*z* 115) and six additional mass units (*m*/*z* 119) compared to C_2_H_6_O_4_ (*m*/*z* 113), thus corresponding to ^13^C_2_H_6_O_4_ and C_2_D_6_O_4_, respectively (fig. S2 and table S2). This confirmed that the R_2_O_4_ compound in the self-reaction of CH_3_O_2_ had indeed the sum formula C_2_H_6_O_4_ and that the ion at *m/z* 113 was C_2_H_6_O_4_(H_2_O)H^+^ and not (C_2_H_8_O_5_)H^+^.

C_2_H_6_O_4_ was identified as the Russell tetroxide (isomer #20 in fig. S7) rather than any other isomers #1 to 19, (*32*), first because most of these isomers would be difficult to form from two CH_3_O_2_ molecules. Exceptions are perhaps isomer #8, CH_2_(OOH)-CH_2_OOH and #18, CH_3_OO-CH_2_OOH, which could potentially be formed if the two CH_3_O_2_ would combine through a C─C or a C─O bond. The different isomers were thus compared using the more quantitative argument of stability, the C_2_H_6_O_4_ compound observed in the experiments having a short lifetime (see the next section), typical of an intermediate rather than a stable molecule. The stability of the 20 isomers of C_2_H_6_O_4_ was estimated by calculating the enthalpy change between 2 × CH_3_O_2_ and C_2_H_6_O_4_. The results are given in fig. S8 and clearly show that the tetroxide (isomer #20) is the least stable compound with an enthalpy change of Δ*H* = −53.2 kJ mol^−1^, consistent with an intermediate. Eighteen of the 19 other isomers, including isomers #8 and #18, have Δ*H* at least 170 kJ mol^−1^ lower than that of the tetroxide, indicating stable molecules with a long lifetime. Only isomer #16, the ethylhydrotetroxide C_2_H_5_-OOOOH, has relatively high Δ*H*, ~ −89.3 kJ mol^−1^, and could also have a short lifetime. However, the formation of this compound from two CH_3_O_2_ molecules was ruled out as highly unlikely.

### Kinetics of formation/decomposition for CH_3_OOOOCH_3_

**Fig. 3.**
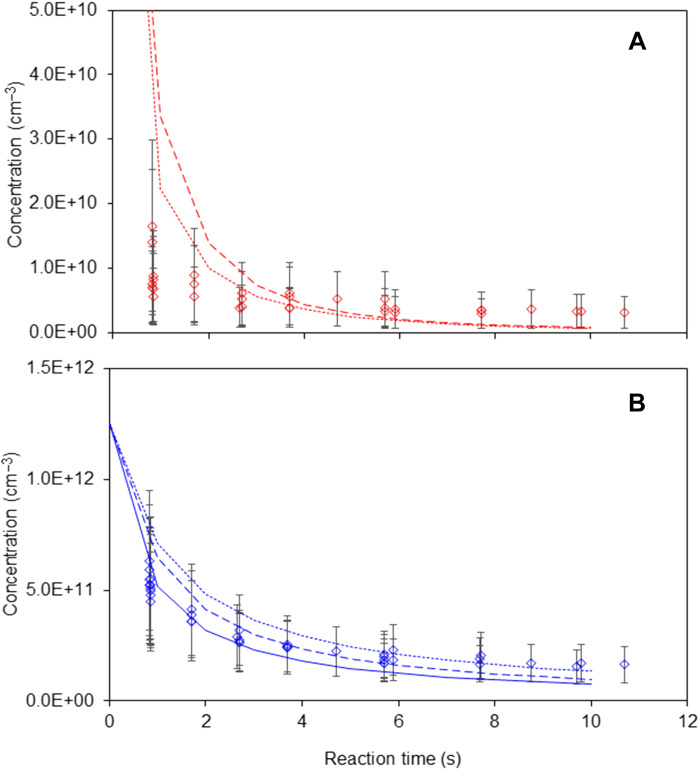
Time profiles and kinetic analysis for CH_3_O_2_ and CH_3_OOOOCH_3_ in the self-reaction of CH_3_O_2_. Experimental data from experiments TETRO19, 20, 21, and 27. (**A**) CH_3_OOOOCH_3_, (**B**) CH_3_O_2_. The solid line in the CH_3_O_2_ profile is the result of the classical second order model not explicating the tetroxide intermediate (model II); The dashed and dotted lines are the results of model I, including the tetroxide, both with *k*_1_/*k*_−1_ = 8 × 10^−14^ cm^3^. The dashed lines are obtained with *k*_1_ = 4 × 10^−10^ cm^−3^ s^−1^, *k*_−1_ = 5 × 10^3^ s^−1^, and *k*_2_ = 4 s^−1^, while the dotted lines are obtained with *k*_1_ = 4 × 10^−13^ cm^−3^ s^−1^, *k*_−1_ = 5 s^−1^, and *k*_2_ = 5 s^−1^, showing that mostly the ratio *k*_1_/*k*_−1_ affects the profiles.

To obtain more information on the tetroxide intermediates from the experiments, the kinetics of formation and decomposition of the tetroxide CH_3_OOOOCH_3_ in the self-reaction of CH_3_O_2_ was studied over 0 to 10 s by combining the data of several experiments (Fig. 3 and fig. S9). These experimental profiles were compared with simulations from a kinetic model involving the formation and decomposition of the tetroxide through Rxns. 1, −1, and 2 (model I in table S3). In addition, the time profile for CH_3_O_2_ was compared to a classical kinetic model of its self-reaction, bypassing the intermediate [model II in table S3 and rate constants from (*33*)]. Using a detection sensitivity determined previously for CH_3_O_2_ (*S^o^*_CH3O2_ ~ 1800 Hz/parts per billion), the classical second-order kinetic model (model II) gave an estimate of the initial concentration of [CH_3_O_2_]_o_ = 1.25 × 10^12^ cm^−3^, which was then applied to model I. The absolute concentration and detection sensitivity for the tetroxide, which were not known, were adapted to the simulation results with the sole constraint that only solutions predicting concentrations above the detection limit (~10^9^ cm^−3^) (*25*) were valid. The values of *k*_1_, *k*_−1_, and *k*_2_ were then varied to fit both the CH_3_O_2_ and tetroxide profiles with model I (Fig. 3 and fig. S9).

Fitting both the CH_3_O_2_ and tetroxide profiles with model I and II proved rather constraining and limited the number of solutions. The results showed that it was the ratio *k*_1_/*k*_−1_, rather than the absolute values of *k*_1_ and *k*_−1_, that affected the kinetic profile for CH_3_OOOOCH_3_, thus suggesting the existence of a preequilibrium between RO_2_ and the tetroxide. The values of *k*_1_ and *k*_−1_ could thus be varied by several orders of magnitudes while providing the same kinetic profile for the tetroxide (Fig. 3 and fig. S9). The range of possible values for *k*_1_ was between 4 × 10^−13^ s^−1^ cm^−3^ (the apparent second-order decay rate for the tetroxide) and 4 × 10^−10^ s^−1^ cm^−3^ (collision limit). Within the uncertainties on the experimental points, the best kinetic fits were obtained with *k*_1_/*k*_−1_ = (5.5 ± 2.5) × 10^−14^ cm^3^ and *k*_2_ = 4 to 10 s^−1^ (Fig. 3 and fig. S9). The tetroxide concentration was found to be between 10^9^ and 3 × 10^10^ cm^−3^, i.e., about 50× lower than that of the RO_2_ over the time profiles. The range of possible values for *k*_−1_ obtained from this analysis implies that the lifetime for the tetroxide CH_3_OOOOCH_3_ ~1/*k*_−1_, is between 0.2 and 200 ms, confirming that it is a reaction intermediate rather than a stable product, thus likely the Russell tetroxide.

### Comparing the kinetic results with theoretical calculations

**Fig. 4.**
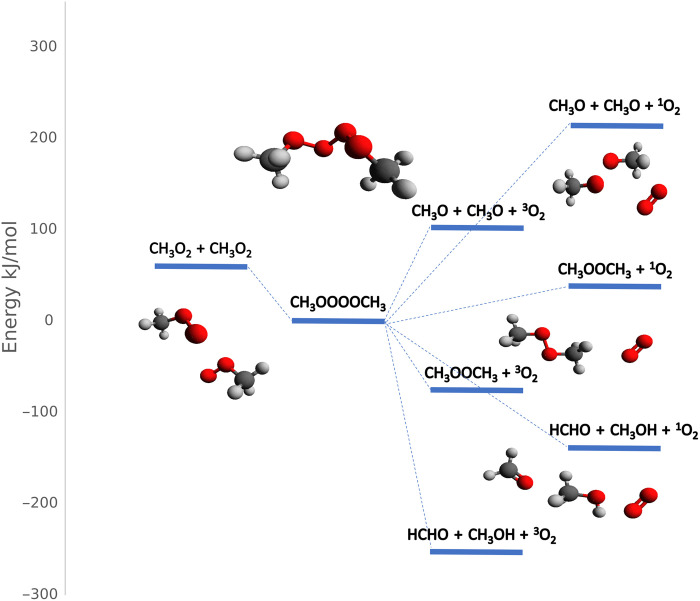
Reaction pathways for the CH_3_O_2_ + CH_3_O_2_ reaction.

The results of the kinetic analysis were compared with theoretical calculations. First, the relative electronic energetics of the various reaction pathways illustrated in Fig. 4 were calculated in ORCA 6.0 (*34*, *35*) at the CCSD(T)/aug-cc-pVTZ//ωB97-D3/aug-cc-pVTZ level along with the possibility of forming O_2_^1^Δ as a product. The results are given in Fig. 4 and show that the CH_3_OOOOCH_3_ intermediate would be expected to be stable with respect to the CH_3_O_2_ reactants by some 60 kJ mol^−1^. In addition, O_2_^1^Δ may be energetically feasible for at least two of the pathways.

The previous kinetic analysis indicated that *k*_2_ ≤ *k*_−1_, which suggests the existence of a preequilibrium between CH_3_OOOOCH_3_ and CH_3_O_2_. The rate coefficients *k*_1_ and *k*_−1_ thus correspond to an equilibrium constant of *K*_eq_ = *k*_1_/*k*_−1_, which can also be expressed in term of statistical mechanics

$$K_{\text{eq}}=\frac{Q_{\text{CH}3\text{OOOOCH}3}}{Q_{\text{CH}3O2}\;Q_{\text{CH}3O2}}e^{\frac{-\Delta H}{\mathrm{RT}}}$$ (Eq. 4)

where *Q*_CH3OOOOCH3_ and *Q*_CH3O2_ are the total partition functions for CH_3_OOOOCH_3_ and CH_3_O_2_ respectively and Δ*H* is the central O─O bond enthalpy (*36*). *K*_eq_ was calculated by determining the partition functions from the vibrational frequencies and rotational constants for CH_3_O_2_ and CH_3_OOOOCH_3_ using various levels of theory and various basis sets in ORCA version 6.0 (Materials and Methods). These partition functions were found to be 1.68 × 10^31^ and 1.39 × 10^34^ cm^−3^ at 300 K for CH_3_O_2_ and CH_3_OOOOCH_3_, respectively (*36*). Theoretical values for the bond enthalpy Δ*H* were calculated from the relative electronic energies, Δ*E*_0_, between 2 CH_3_O_2_ and CH_3_OOOOCH_3_ using coupled-cluster (CC) methods in ORCA (Materials and Methods). In total, seven calculations were performed with different basis sets and the results are given in table S5. The values of 59 to 62 kJ mol^−1^ obtained for the relative electronic energies, Δ*E*_0_, agree closely with those of Salo *et al.* (*16*) using similar methods. The corresponding bond enthalpy values, Δ*H*, derived from the difference between the various calculated electronic energies for CH_3_O_2_ and CH_3_OOOOCH_3_ using zero-point corrections from the density functional theory (DFT) calculations give a value of ~49 kJ mol^−1^. Combining this bond enthalpy with the previously calculated partition function leads to an equilibrium constant of *K*_eq_ = 2.25 × 10^−20^ cm^3^, which is considerably lower than the value determined experimentally.

Similar discrepancies have been noted for the tetroxide intermediate in the HO_2_ + HO_2_ reaction (*37*). In that case, it was found necessary to reduce the vibrational frequencies of the tetroxide intermediate to match the experimental data and capture the observed pressure and temperature dependence of the reaction. This is presumably due to the presence of very low frequency modes or internal rotors in the loose, floppy intermediate molecule. Therefore, in this case, the lowest five vibrational frequencies in CH_3_OOOOCH_3_ were replaced by internal rotors and the other frequencies were reduced by an anharmonic scale factor of 0.9612 in line with standard practice for DFT calculated vibrational frequencies (*38*). More details are given in Materials and Methods. The net result was to increase *Q*_CH3OOOOCH3_ to 1.57 × 10^38^ cm^−3^ giving an equilibrium constant of 5.5 × 10^−14^ cm^3^ consistent with the experimental findings.

## DISCUSSION

### Implications for the understanding of radical reactions in aerobic systems

This work reports the first systematic observation of symmetric tetroxides (methyl-, ethyl-, and isopropyl tetroxides) and cross-tetroxides in a range of RO_2_ reactions since the Russell postulate of 1957. The existence of these tetroxides has been assumed for decades despite the lack of direct evidence, and directly observing them in this work confirms the main feature of the Russell mechanism. Not only this work establishes that these tetroxides are relatively stable at room temperature in the gas phase but also that the range of lifetime determined for the methyl tetroxide is consistent with that of intermediates. The experimental kinetic results for the methyl tetroxide match the theoretical expectations if this intermediate is assumed to be a loose molecule with low vibrational frequencies and internal rotations. These experimental results can be useful to constrain theoretical studies of these intermediates. This work thus shows that gas-phase studies might be a more convenient experimental approach than condensed-phase ones for the investigation of the Russell mechanism. Gas-phase studies might thus be relevant to investigate radical reactions in other fields of applications such as biochemistry and medicine.

The results of this work indicate that, in room-temperature gas-phase aerobic systems, such as in Earth’s atmosphere, the concentration of tetroxides could be as high as ~1/50 times that of the corresponding RO_2_. Further works will need to explore their potential other reactions, such as their photolysis by sunlight, to determine their exact concentration. However, other compounds containing more than two consecutive O-atoms, hydrotrioxides, ROOOH, were estimated to be present in nonnegligible quantities in the atmosphere (*39*).

Observing the tetroxide intermediates was made possible in this work by the optimization of a direct mass spectrometric technique toward the speciated observation of radicals and other elusive compounds. This development is part of a general trend in the development of direct mass spectrometric instruments, allowing today to detect compounds that were not possible to observe over a decade ago, such as highly oxygenated molecules and radicals (*7*), peroxy radicals (*24*, *25*), and even hydrotrioxides (*39*). The technique used in this work could thus be used to study other intermediates, which would improve the understanding of organic oxidation in aerobic systems and their many applications.

## MATERIALS AND METHODS

### Experimental design

The experimental setup has been described in previous works (*40*). The experiments were performed in vertical quartz reactors of total length *L* = 120 cm and internal diameters, *d* = 3 and 5 cm. Organic peroxy radicals, RO_2_, were produced in the reactors photolytically by flowing a gas mixture containing the radical precursor through an irradiation window, corresponding to 2 to 8 s of residence time. This irradiation window was surrounded by 4 narrow-band UV-C lamps (Phillips TUV 36 W SLV/6) emitting at λ = 254 nm. After the irradiation window, the gas mixture was flown in the dark, to allow for further reactions to take place over a reaction time between 0.5 and 10 s, which was varied by moving the position of the irradiation window. At the outflow of the reactor, a small fraction (<10%) of the gas flow was sampled into a chemical ionization mass spectrometer for analysis. The bath gas was synthetic air with a mass flow of 3.0 sLm, and the experiments were performed slightly below atmospheric pressure (*P* = 0.85 to 0.95 atm) and at room temperature (*T* = 300 ± 4 K). The radical precursors were introduced into the main gas flow by bubbling a small flow of N_2_ through the pure liquids and adding them to the reactor after a dilution loop. A list of the experiments performed in this work is given in table S1.

### Radical production

The radicals studied in this work, CH_3_O_2_, ^13^CH_3_O_2_, CD_3_O_2_, C_2_H_5_O_2_, and i-C_3_H_7_O_2_, were generated by photolyzing their respective iodinated precursors: CH_3_I, ^13^CH_3_I, CD_3_I, C_2_H_5_I, and 2-C_3_H_7_I. The radical production proceeded by the sequence of reactions illustrated below for CH_3_O_2_

$${\text{CH}}_{3}I+\mathrm{h\nu}\to {\text{CH}}_{3}+I$$ (Rxn. 5)

$${\text{CH}}_{3}+O_{2}+M\to {\text{CH}}_{3}O_{2}+M$$ (Rxn. 6)

On the basis of recent calibrations under the same ionization conditions (*40*), the RO_2_ concentration in the experiments was estimated between 1 and 4 × 10^12^ cm^−3^ for all RO_2_ except i-C_3_H_7_O_2_, which was estimated of the order of 10^13^ cm^−3^.

### Tetroxides detection

All the compounds present in the reactor outflow were ionized by proton transfer with proton water clusters, (H_2_O)_n_H^+^ as described by Eq. 3 above. Under the ionization conditions used in this study, the most abundant water/proton clusters were (H_2_O)_3_H^+^ (*m/z* 55) and (H_2_O)_4_H^+^ (*m/z* 73) rather than H_3_O^+^. Thus, a compound A of mass M was almost exclusively detected by its ion water clusters at *m/z* M + 19 [A(H_2_O)H^+^], M + 37 [A(H_2_O)_2_H^+^], and even in some cases M + 55[“A(H_2_O)_3_H^+^”]. The ion masses at which the radicals and products were detected in this study are listed in table S2.

### Theoretical methods

The vibrational frequencies and rotational constants for CH_3_OOOOCH_3_ and CH_3_O_2_ were calculated using various levels of theory and various basis sets using ORCA version 6.0 (*34*, *35*). DFT geometry optimizations and frequency calculations were carried out using ωB97W-D3 and M06-2X functionals with the fully augmented, triple-ζ correlation consistent basis set, aug-cc-pVTZ (*41*, *42*). The optimized structure was a staggered linear chain of four O atoms with a CH_3_ group at each end and the resulting vibrational frequencies and rotational constants are given in table S4 for the ωB97W-D3/aug-cc-pVTZ case. The M06-2X functional gave almost identical results and are not shown.

The central O─O bond enthalpy for CH_3_OOOOCH_3_ was calculated theoretically using the CCSD(T) method in ORCA 6.0 with CC singles and doubles with perturbative triples. The CCSD(T) calculations were done with aug-cc-pVDZ, aug-cc-pVTZ, and aug-cc-pVQZ basis sets (*43*) with complete basis set (CBS) limit extrapolation (*44*–*46*). Two-point extrapolations using either aug-cc-pVDZ and aug-cc-pVTZ or aug-cc-pVTZ and aug-cc-pVQZ were used to obtain CBS energies, denoted as CBS (2/3) or CBS (3/4). The results of these calculations are given in table S5 and the values for the relative electronic energies, Δ*E*_0_, of 2 CH_3_O_2_ and CH_3_OOOOCH_3_ agree closely with those of Salo *et al.* (*16*) using similar methods. Bond enthalpy, Δ*H*, values were derived from the difference between the various calculated electronic energies for CH_3_O_2_ and CH_3_OOOOCH_3_ using zero-point corrections from the ωB97W-D3/aug-cc-pVTZ calculations.
