# Supplementary material for: Observing elusive tetroxides in gas-phase radical reactions supports the Russell mechanism
Source: Sci Adv. 2026 Mar 13;12(11):eaeb6495. doi: 10.1126/sciadv.aeb6495 (PMC12985734; doi:10.1126/sciadv.aeb6495)
Supplement: Supplementary file 1 — Figs. S1 to S9 Tables S1 to S5 [file sciadv.aeb6495_sm.pdf]

Science Advances
AAAS

Supplementary Materials for

Observing elusive tetroxides in gas-phase radical reactions supports the Russell mechanism

Barbara Nozière and Roger Patrick

Corresponding author: Barbara Noziere, noziere@kth.se

Sci. Adv. **12**, eaeb6495 (2026)
DOI: 10.1126/sciadv.aeb6495

This PDF file includes:

Figs. S1 to S9
Tables S1 to S5

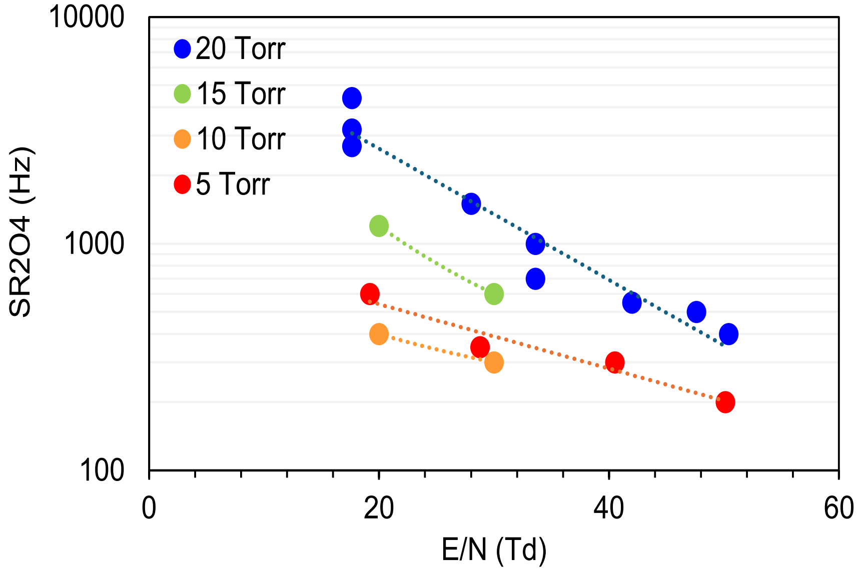

Fig. S1. Effects of the ionization conditions on the detection of R_2O_4 ions. Effects of the ionization conditions on the detection of the $C_2H_6O_4(H_2O)H^+$ (*m/z* 113) and $C_2H_6O_4(H_2O)_2H^+$ (*m/z* 131) ions. The signals reported in this graph are the sum of the signals at *m/z* 113 and 131.

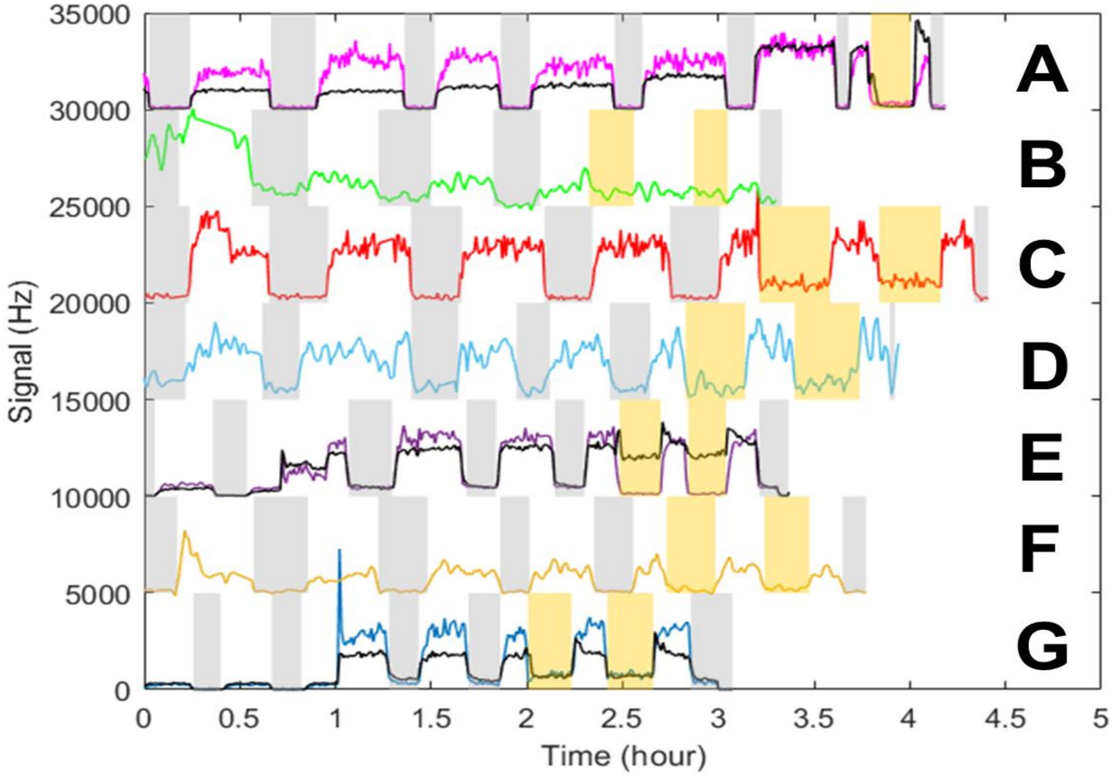

Fig. S2. Time profiles for the tetroxides in the self- and cross-reactions of different RO_2. A) self-reaction of CH_3O_2. Black: CH_3O_2 (× 0.2), pink: $C_2H_6O_4$ (× 2); B) $^{13}C_2H_6O_4$ in the self-reaction of $^{13}CH_3O_2$ (× 15); C) $C_2D_6O_4$ in the self-reaction of CD_3O_2 (× 2); D) $C_4H_{10}O_4$ (× 20) in the self-reaction of $C_2H_5O_2$; E) Cross-reaction between $C_2H_5O_2$ and CH_3O_2. Black: CH_3O_2 (× 0.1), purple: $C_2H_5O_4CH_3$ (× 0.5); F) i-$C_6H_{14}O_4$ (× 5) in the self-reaction of i-$C_3H_7O_2$; G) Cross-reaction between i-$C_3H_7O_2$ and CH_3O_2. Black: CH_3O_2 (× 0.1), blue: i-$C_3H_7O_4CH_3$ (× 0.5). In all cases, the tetroxide signal disappears when the lights are OFF (no RO_2 produced, grey areas) and when NO is added to the reactor because the reaction RO_2+NO consumes the RO_2 and takes over the RO_2 self- and cross-reactions (no tetroxides produced. Orange areas). Note that, in the self-reaction of $^{13}CH_3O_2$ (B) the decrease of the R_2O_4 signal when adding NO is cancelled out by the formation of $^{13}CH_3ONO_2$, which is an isobar of the tetroxide (*m/z* 97 + 115) .

Fig. S3.

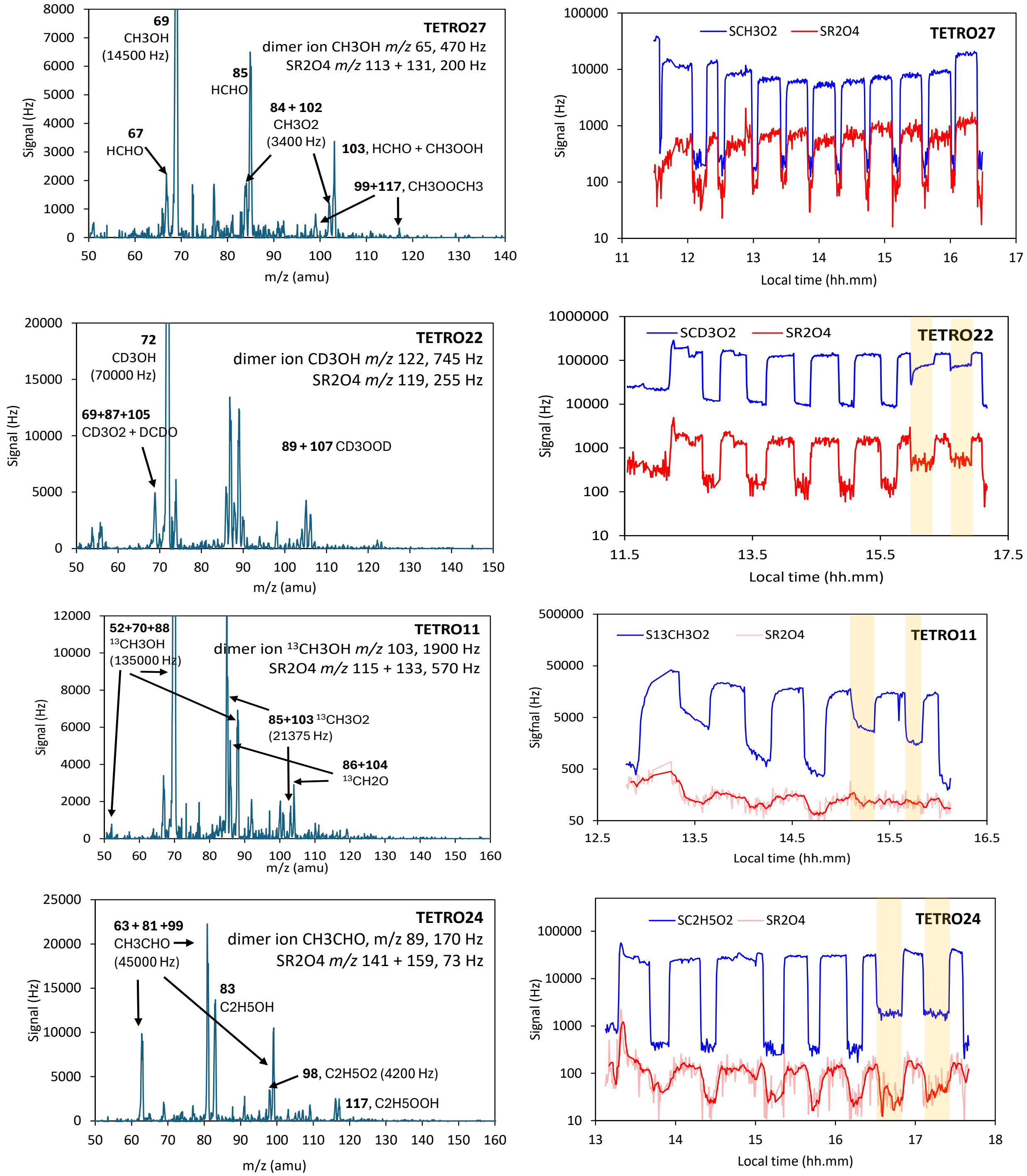
TETRO27
dimer ion CH3OH m/z 65, 470 Hz
SR2O4 m/z 113 + 131, 200 Hz
69
CH3OH
(14500 Hz)
85
HCHO
67
HCHO
84 + 102
CH3O2
(3400 Hz)
103, HCHO + CH3OOH
99+117, CH3OOCH3
Signal (Hz)
m/z (amu)
SCH3O2
SR2O4
TETRO27
Signal (Hz)
Local time (hh.mm)
TETRO22
dimer ion CD3OH m/z 122, 745 Hz
SR2O4 m/z 119, 255 Hz
72
CD3OH
(70000 Hz)
69+87+105
CD3O2 + DCDO
89 + 107 CD3OOD
Signal (Hz)
m/z (amu)
SCD3O2
SR2O4
TETRO22
Signal (Hz)
Local time (hh.mm)
TETRO11
dimer ion 13CH3OH m/z 103, 1900 Hz
SR2O4 m/z 115 + 133, 570 Hz
52+70+88
13CH3OH
(135000 Hz)
85+103 13CH3O2
(21375 Hz)
86+104
13CH2O
Signal (Hz)
m/z (amu)
S13CH3O2
SR2O4
TETRO11
Sigfnal (Hz)
Local time (hh.mm)
TETRO24
dimer ion CH3CHO, m/z 89, 170 Hz
SR2O4 m/z 141 + 159, 73 Hz
63 + 81 +99
CH3CHO
(45000 Hz)
83
C2H5OH
98, C2H5O2 (4200 Hz)
117, C2H5OOH
Signal (Hz)
m/z (amu)
SC2H5O2
SR2O4
TETRO24
Signal (Hz)
Local time (hh.mm)

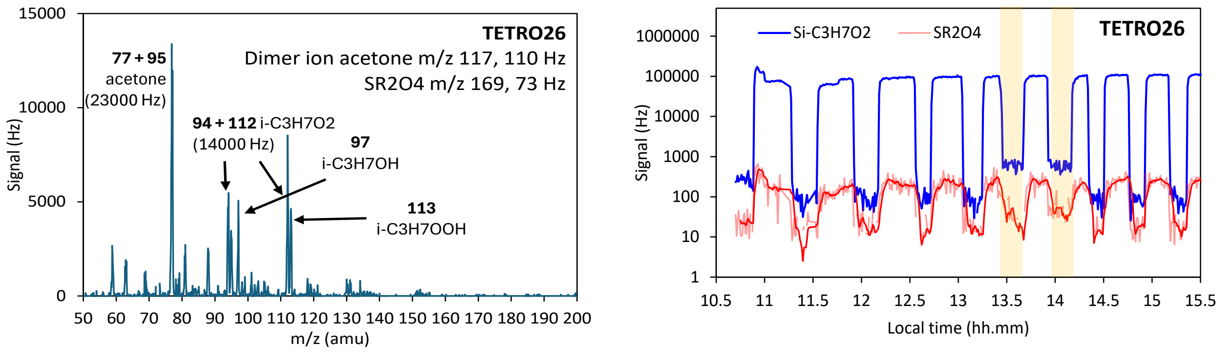

Fig. S3. Examples of spectra and time profiles obtained in the self-reactions of RO2. Each mass spectrum identifies the ions for the main reaction products, radical, and tetroxide. Signal intensities are also provided for the most intense ion (main reaction product), its potential dimer ion, and for the observed tetroxide ion. In the time profiles, the periodic signal decrease correspond to cycles when the photolysis lamps were turned Off (no RO2 produced, thus no tetroxide). The areas marked in orange are those where NO was added to the reactor (RO_2 self-reaction replaced by RO_2 + NO, thus no tetroxide produced). All the time profile signals are normalized for 1 MHz of total proton water clusters signal, which accounts for the larger intensities in the time profiles than in the corresponding spectra.

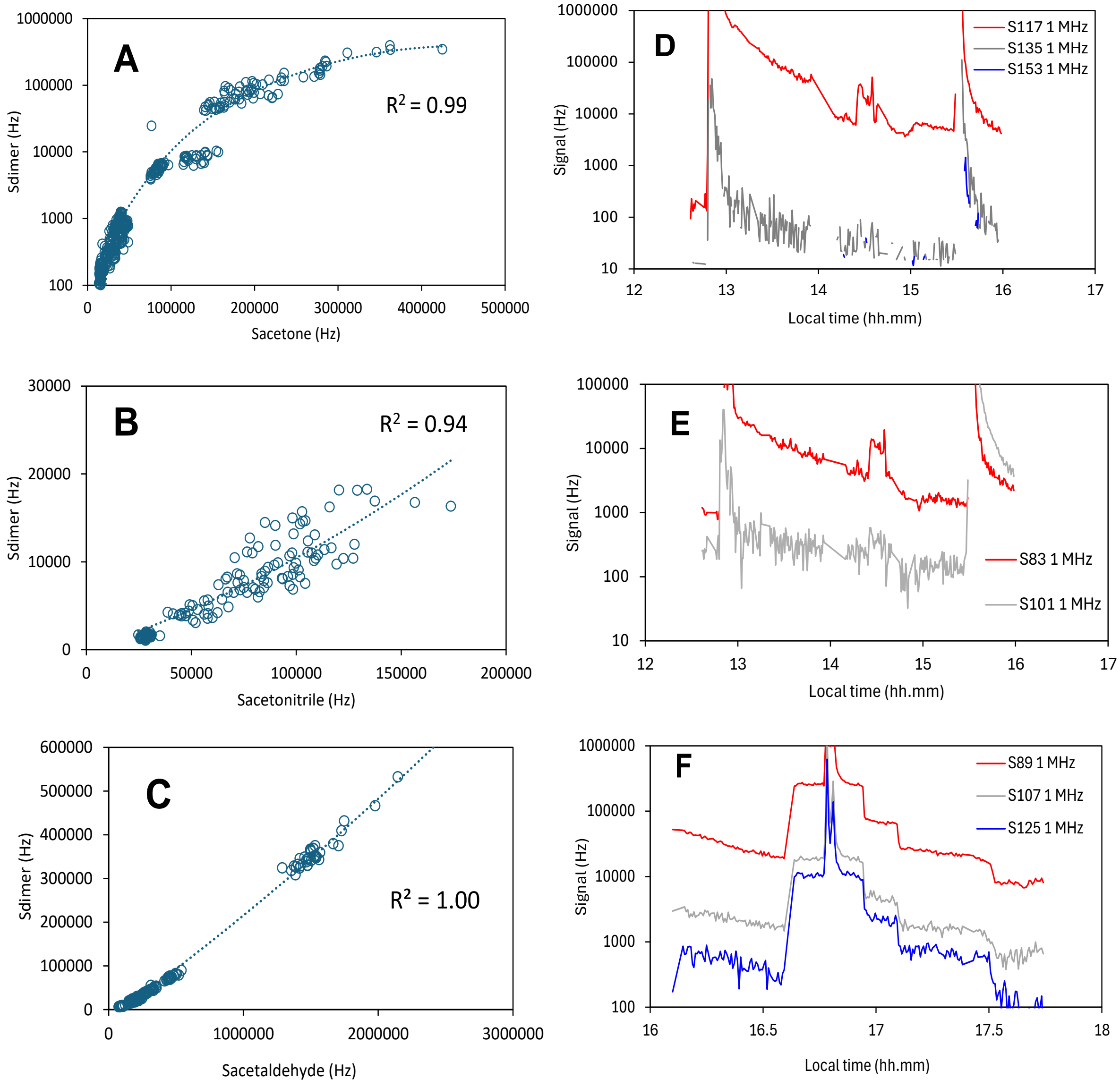

Fig. S4. Dimer ion formation from acetone, acetonitrile, and acetaldehyde. A - C: Evolution of the dimer signal as function of the monomer signal. The dotted lines show the second order variation. C - E: relative signals of the ion water clusters for the dimers, showing that the ion M+1 (“AH^+”) is at least an order of magnitude more abundant than the water cluster ions M+19 (“$A(H_2O)H^+$”) and M+37 (“$A(H_2O)_2H^+$”): M+1: red lines, M+19: grey lines, M+37: blue lines.

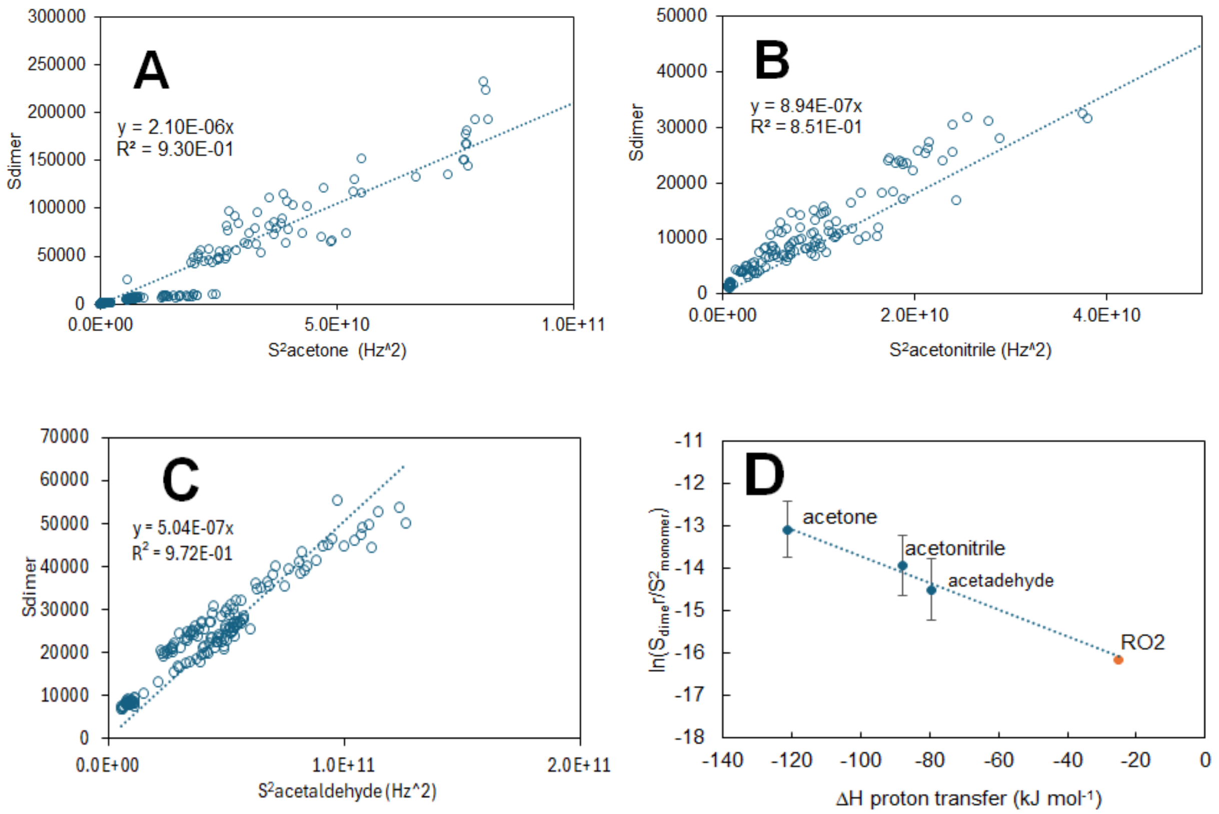

Fig. S5. Comparing ion dimerization between different compounds. A)-C): Determination of the signal ratios $S_{dimer}/S^2_{monomer}$ in the ion dimerization of acetone, acetonitrile and acetaldehyde. D) Correlations between these ratios and ΔH (kJ mol^{-1}) for the proton transfer reaction of the respective compounds and extrapolation to RO_2. The error bars represent +/- 5 % of uncertainties on each value of $\ln(S_{dimer}/S^2_{monomer})$.

Fig. S6.

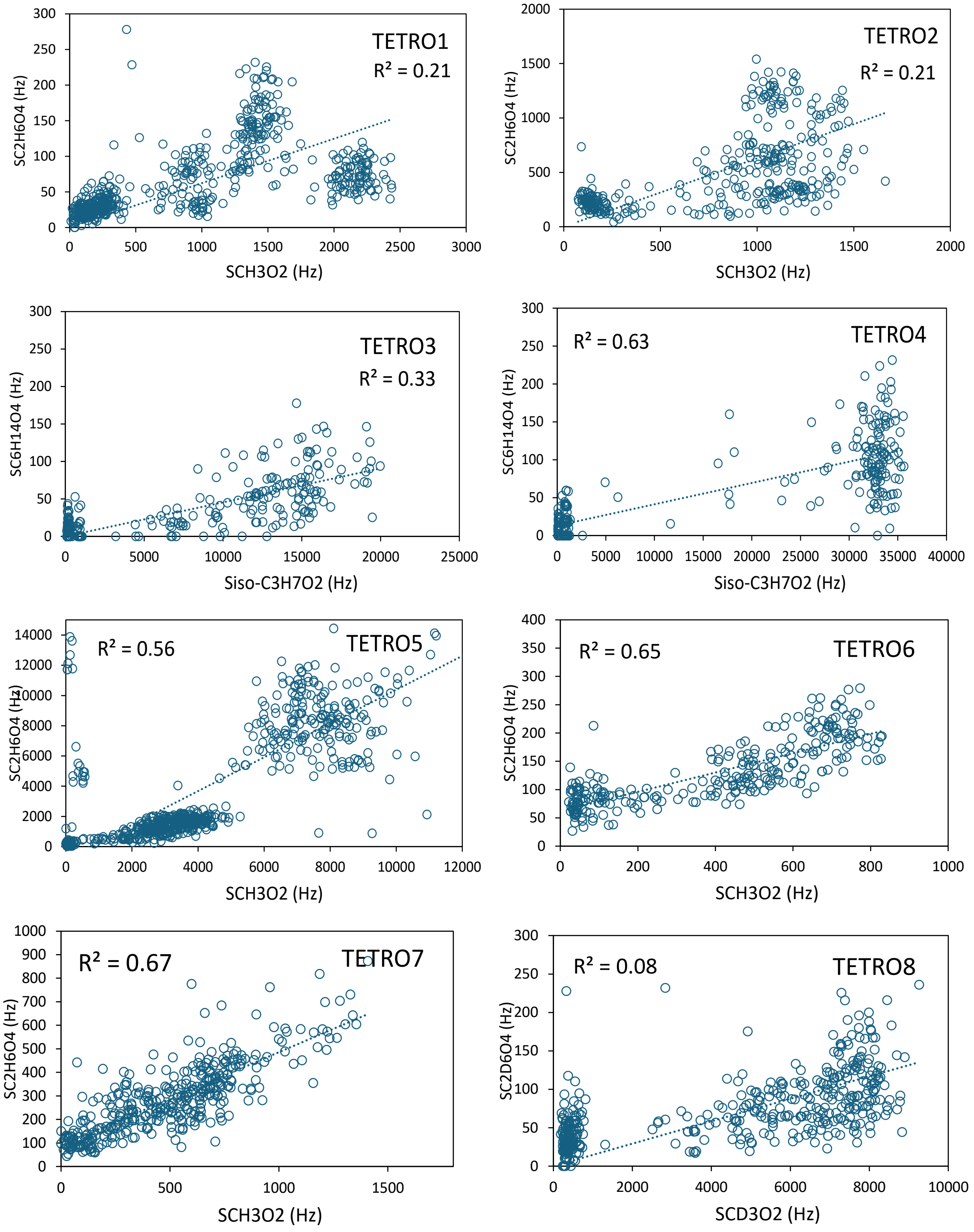

TETRO1
R² = 0.21
SC2H6O4 (Hz)
SCH3O2 (Hz)
TETRO2
R² = 0.21
SC2H6O4 (Hz)
SCH3O2 (Hz)
TETRO3
R² = 0.33
SC6H14O4 (Hz)
Siso-C3H7O2 (Hz)
R² = 0.63
TETRO4
SC6H14O4 (Hz)
Siso-C3H7O2 (Hz)
R² = 0.56
TETRO5
SC2H6O4 (Hz)
SCH3O2 (Hz)
R² = 0.65
TETRO6
SC2H6O4 (Hz)
SCH3O2 (Hz)
R² = 0.67
TETRO7
SC2H6O4 (Hz)
SCH3O2 (Hz)
R² = 0.08
TETRO8
SC2D6O4 (Hz)
SCD3O2 (Hz)

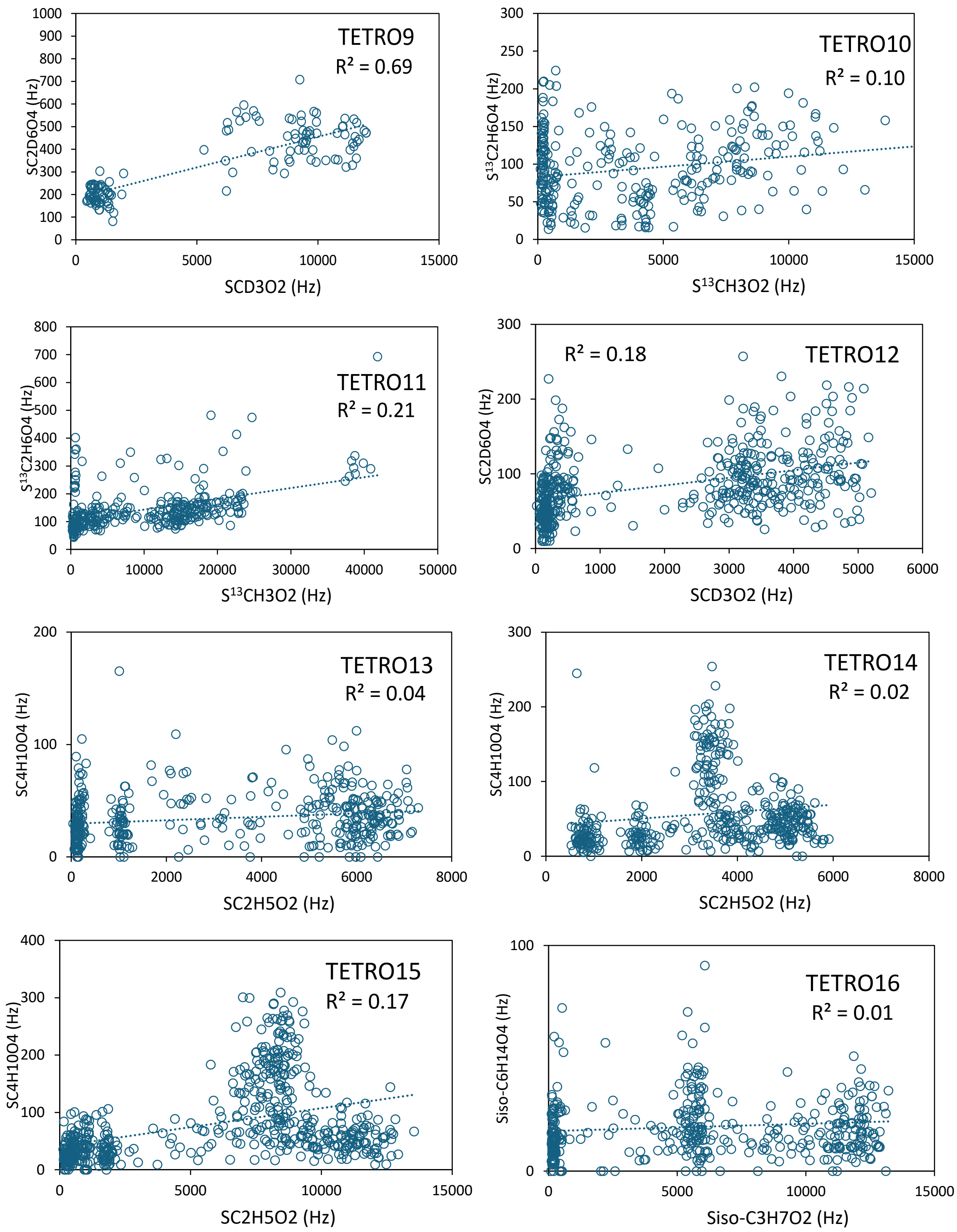

TETRO9
R² = 0.69
SC2D6O4 (Hz)
SCD3O2 (Hz)
TETRO10
R² = 0.10
S13C2H6O4 (Hz)
S13CH3O2 (Hz)
TETRO11
R² = 0.21
S13C2H6O4 (Hz)
S13CH3O2 (Hz)
R² = 0.18
TETRO12
SC2D6O4 (Hz)
SCD3O2 (Hz)
TETRO13
R² = 0.04
SC4H10O4 (Hz)
SC2H5O2 (Hz)
TETRO14
R² = 0.02
SC4H10O4 (Hz)
SC2H5O2 (Hz)
TETRO15
R² = 0.17
SC4H10O4 (Hz)
SC2H5O2 (Hz)
TETRO16
R² = 0.01
Siso-C6H14O4 (Hz)
Siso-C3H7O2 (Hz)

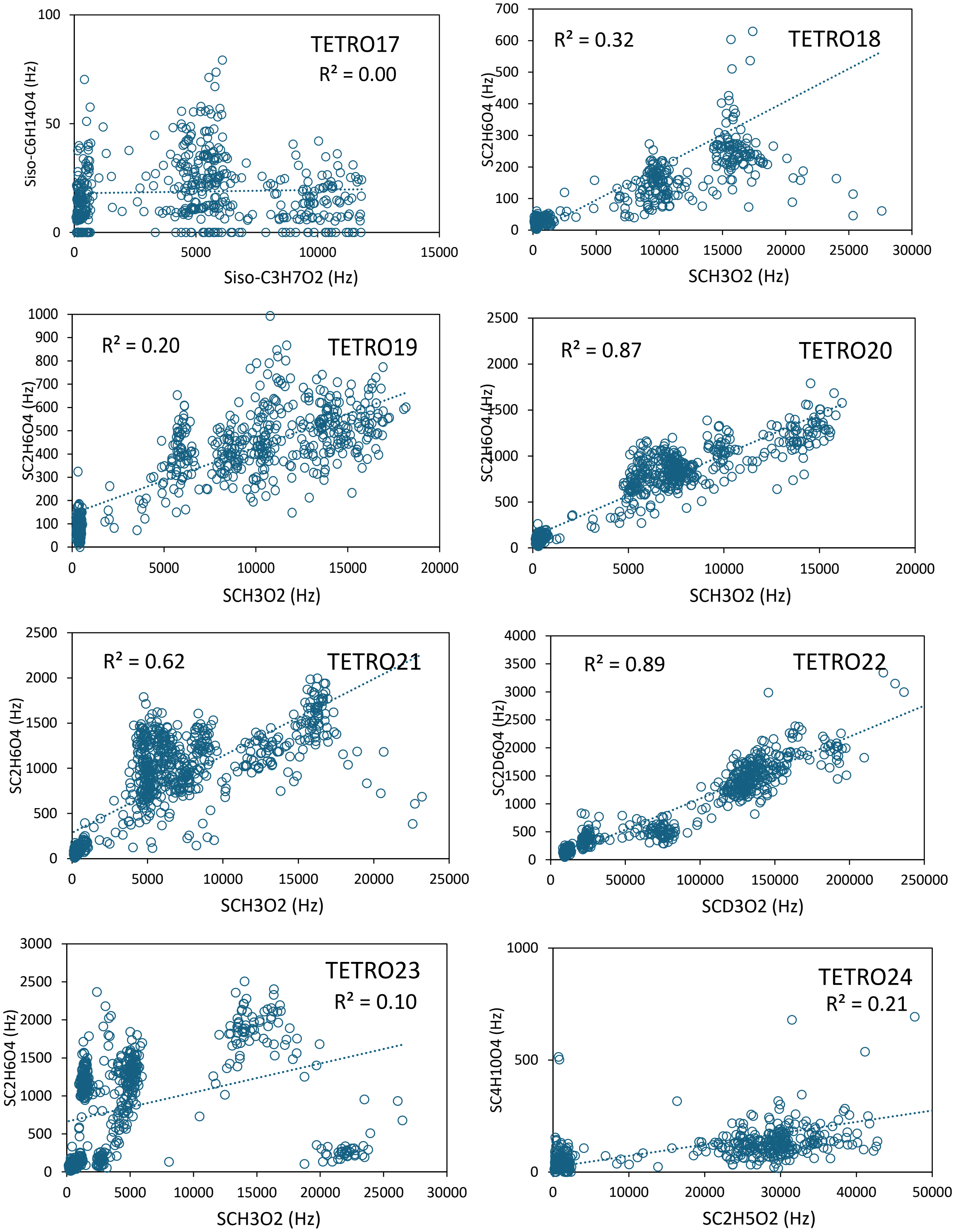

TETRO17
R² = 0.00
Siso-C6H14O4 (Hz)
Siso-C3H7O2 (Hz)
100
50
0
0
5000
10000
15000
TETRO18
R² = 0.32
SC2H6O4 (Hz)
SCH3O2 (Hz)
700
600
500
400
300
200
100
0
0
5000
10000
15000
20000
25000
30000
TETRO19
R² = 0.20
SC2H6O4 (Hz)
SCH3O2 (Hz)
1000
900
800
700
600
500
400
300
200
100
0
0
5000
10000
15000
20000
TETRO20
R² = 0.87
SC2H6O4 (Hz)
SCH3O2 (Hz)
2500
2000
1500
1000
500
0
0
5000
10000
15000
20000
TETRO21
R² = 0.62
SC2H6O4 (Hz)
SCH3O2 (Hz)
2500
2000
1500
1000
500
0
0
5000
10000
15000
20000
25000
TETRO22
R² = 0.89
SC2D6O4 (Hz)
SCD3O2 (Hz)
4000
3500
3000
2500
2000
1500
1000
500
0
0
50000
100000
150000
200000
250000
TETRO23
R² = 0.10
SC2H6O4 (Hz)
SCH3O2 (Hz)
3000
2500
2000
1500
1000
500
0
0
5000
10000
15000
20000
25000
30000
TETRO24
R² = 0.21
SC4H10O4 (Hz)
SC2H5O2 (Hz)
1000
500
0
0
10000
20000
30000
40000
50000

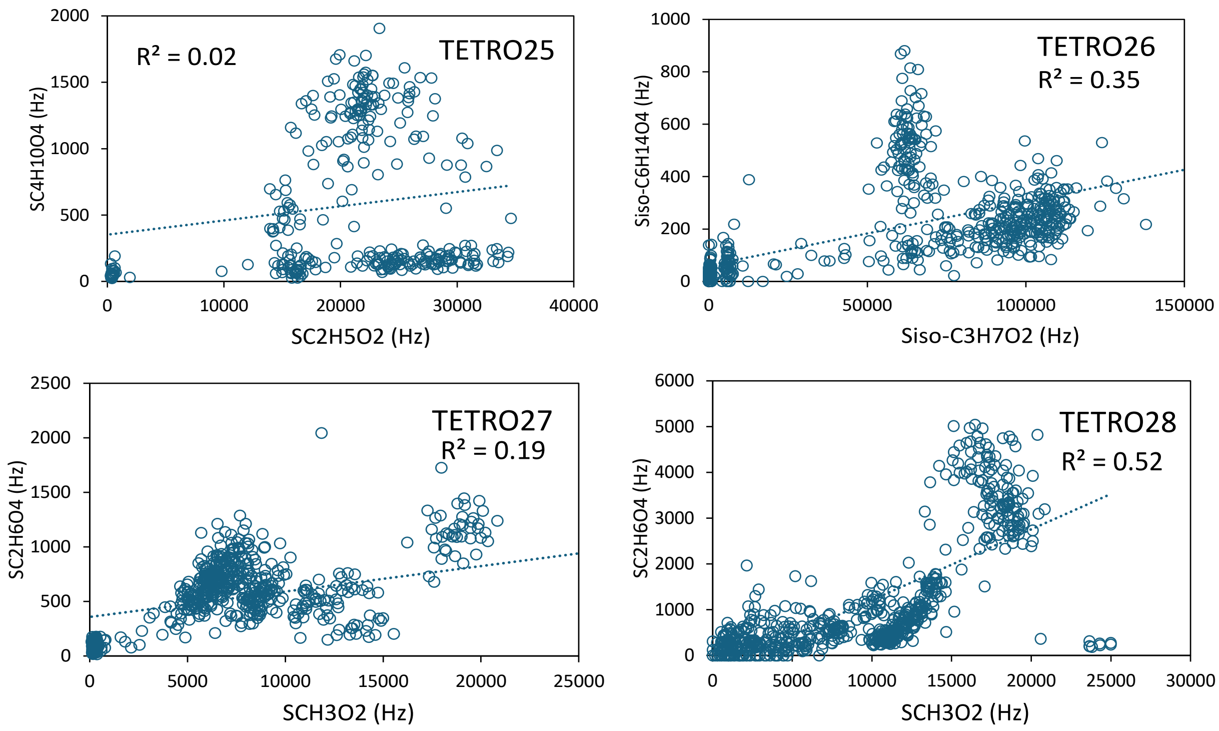

Fig. S6. Correlations between the $R_2O_4(H_2O)H^+$ and RO_2 signals in the experiments

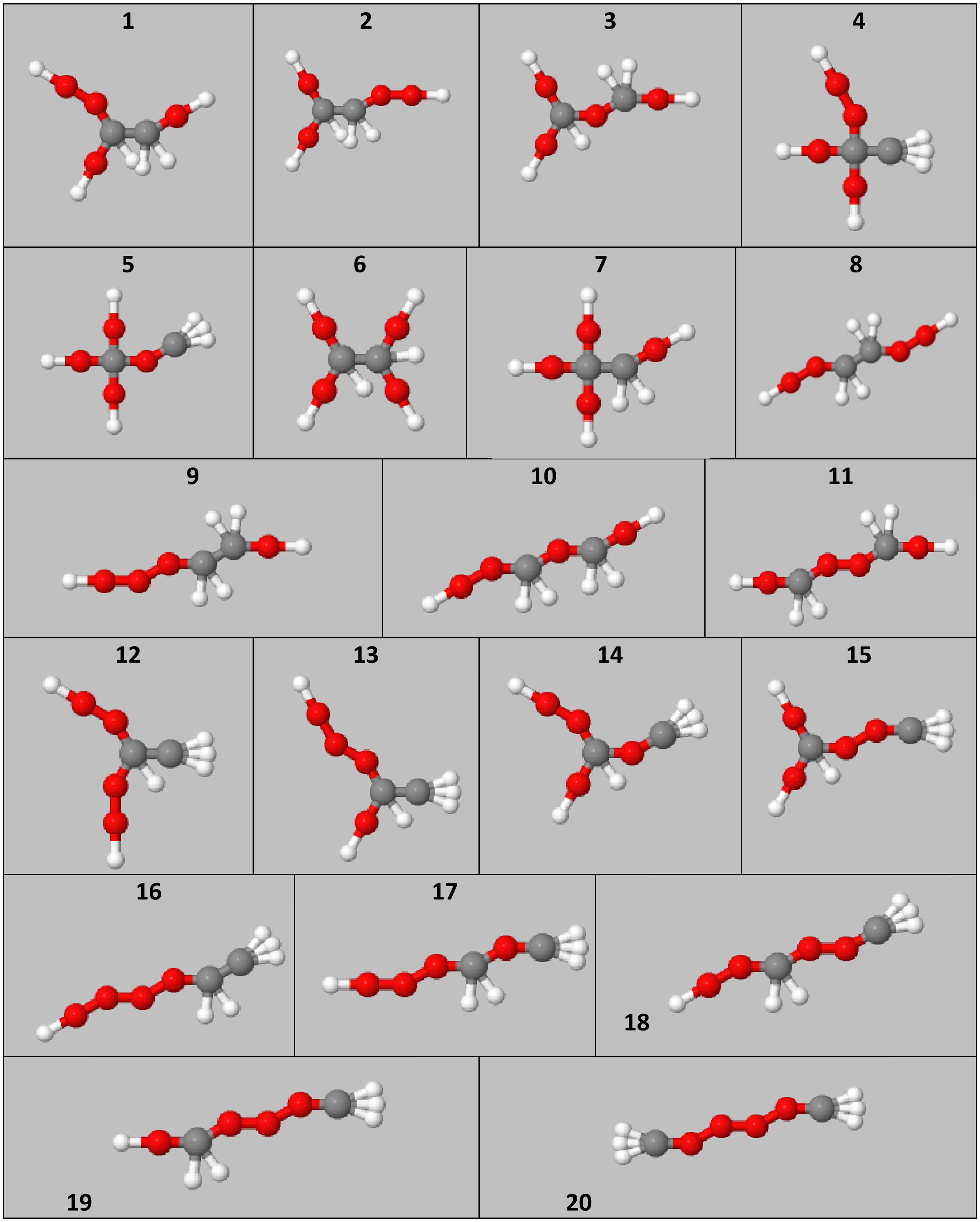

Fig. S7. List and structures of the isomers for $C_2H_6O_4$ from ref. *(32)*

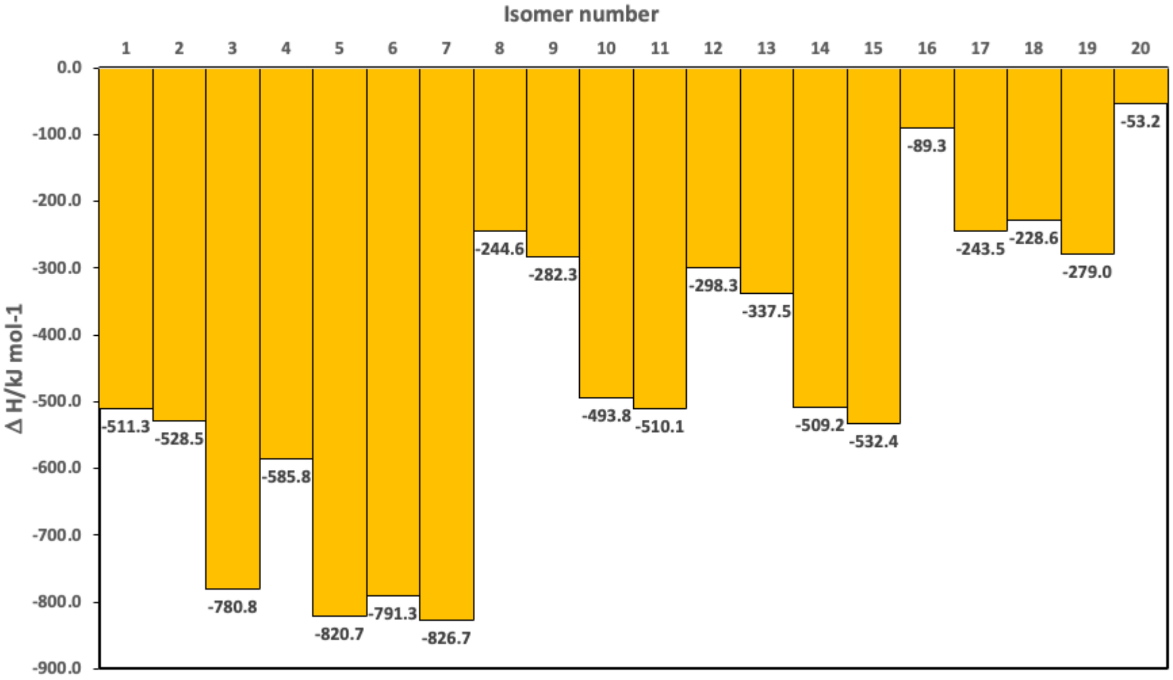

Fig. S8. Quantum calculations of the stability of the 20 isomers for $C_2H_6O_4$. The stability for each isomer was calculated as the enthalpy change for the reaction 2 $CH_3O_2 \rightarrow C_2H_6O_4$ at the CCSD(T)/aug-cc-pVTZ//ωB97X-D3/aug-cc-pVTZ level using ORCA 6.0.*(34)*

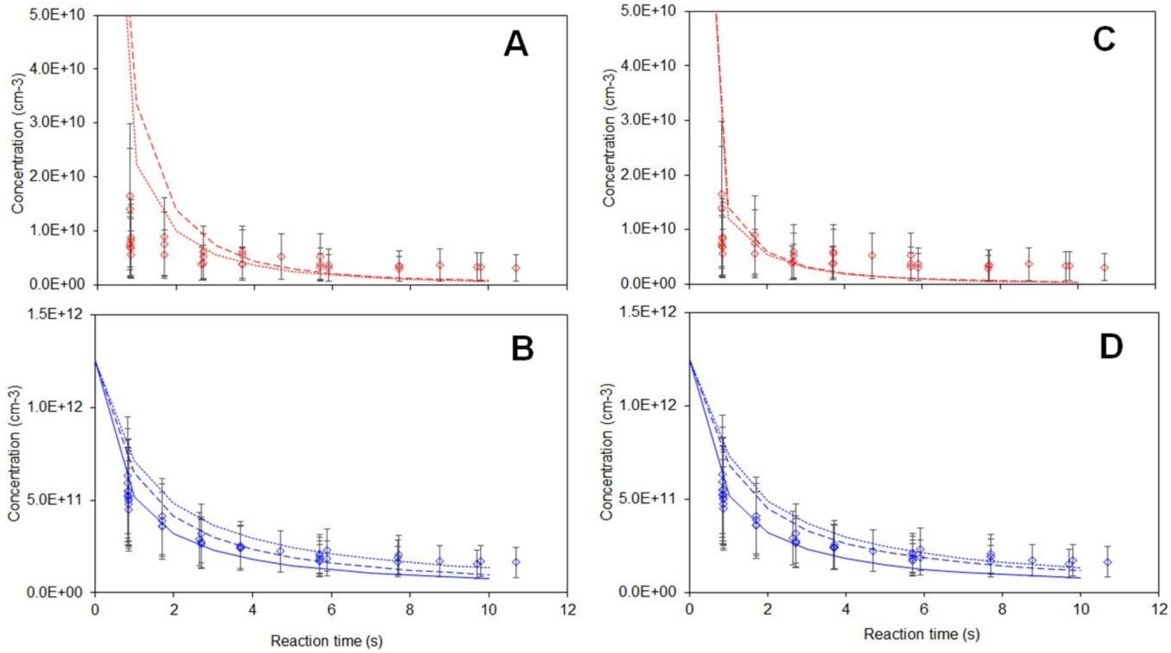

Fig. S9. Results of the kinetic analysis. In blue: CH_3O_2, in red: $CH_3OOOOCH_3$. Comparison of the curves obtained with $k_1/k_{-1} = 8 \times 10^{-14}$ cm^3 and $k_1 = 4 \times 10^{-13}$ and 4×10^{-10} cm^3 s^{-1} (A and B as in Fig. 3) with those obtained with $k_1/k_{-1} = 4 \times 10^{-14}$ cm^3 and $k_1 = 1 \times 10^{-12}$ cm^3 s^{-1}, $k_{-1} = 25$ s^{-1} and $k_2 = 9$ s^{-1} (C and D dashed lines) and with $k_1/k_{-1} = 3 \times 10^{-14}$ cm^3 and $k_1 = 1 \times 10^{-12}$ cm^3 s^{-1}, k_{-1} = 35 s^{-1} and $k_2 = 10$ s^{-1} (C and D dotted lines). As in Fig.3 the solid line in the profiles of CH_3O_2 is the simulation obtained from the classical second-order kinetics.

Table S1. List of the experiments and conditions.

Expt#	RO_2	[RI] (ppm)	Drift tube pressure (Torr)	Electrical energy E/N (Td)
TETRO1	CH3O2	12.9	15.0	23.7
TETRO2	CH3O2	12.9 - 26	18.0	19.8
TETRO3	C3H7O2 + CH3O2	7 /3.5	19.0	19.3
TETRO4	C3H7O2 + CH3O2	7 / 8.6	21.0	17.5
TETRO5	CH3O2	17	19.0	20.8
TETRO6	CH3O2	26	19.3	18.5
TETRO7	CH3O2	52	20.0	18.1
TETRO8	CD3O2	104*	20.1	17.8
TETRO9	CD3O2	87*	20.2	17.7
TETRO10	^{13}CH3O2	87*	20.2	17.7
TETRO11	^{13}CH3O2	58*	20.3	17.5
TETRO12	CD3O2	69*	19.7	18.1
TETRO13	C2H5O2	44.5	20.0	26.8
TETRO14	C2H5O2 + CH3O2	44.5/132.0	20.6	17.4
TETRO15	C2H5O2 + CH3O2	44.5/132.0	20.0	17.9
TETRO16	i-C3H7O2 + CH3O2	10.7/69.0	20.4	17.5
TETRO17	i-C3H7O2 + CH3O2	25.5/34.1	20.1	17.8
TETRO18	CH3O2	11.4	20.5	17.5
TETRO19	CH3O2	11.4	20.0	17.8
TETRO20	CH3O2	13.7	20.5	17.4
TETRO21	CH3O2	13.7	20.3	17.5
TETRO22	CD3O2	34.0*	20.7	17.2
TETRO23	CH3O2	13.7	20.3	17.5
TETRO24	C2H5O2	5.8	20.1	17.8
TETRO25	C2H5O2+CH3O2	5.8/17.1	20.4	17.6
TETRO26	iC3H7O2+CH3O2	10.5/67.2	19.7	18.1
TETRO27	CH3O2	13.7	20.4	17.5
TETRO28	CH3O2	17.1	20.3	17.5

*Estimated assuming the same vapor pressure for $^{13}CH_3I$ and CD_3I than for CH_3I.

Table S2. Ion masses at which the compounds were observed.

In bold are the main ions observed. Under the ionization conditions used in this study, the proton transfer ionization of the compounds present in the reactor led to the corresponding ion water clusters M+19 (i.e. $A(H_2O)H^+$ ions) and M+37 ($A(H_2O)_2H^+$), while the ion water cluster M+1 (AH^+) was not observed.

RO_2 (MW)	RO_2 observed *m/z*	ROOOOR (MW)	ROOOOR Observed *m/z*
CH_3O_2 (47)	**84/102**	$C_2H_6O_4$ (94)	**113/**131
CD_3O_2 (50)	69/**87/**105	$C_2D_6O_4$ (100)	**119/**137
$^{13}CH_3O_2$ (48)	**85/**103	$^{13}C_2H_6O_4$ (96)	**115/**133
$H_3C\text{-}CH_2O_2$ (61)	80/**98/**116	$C_4H_{10}O_4$ (122)	**141/**159
cross reaction w. CH_3O_2		$C_3H_8O_4$ (108)	**127/**145*
$H_3C\text{-}CHO_2\text{-}CH_3$ (75)	94/**112**	$C_6H_{14}O_4$ (150)	**169/**187
cross reaction w CH_3O_2		$C_4H_{10}O_4$ (122)	**123/**141
	Monomers	Dimer ions	
Acetone (58)	59/**77/**95	**117/**135/153	
Acetonitrile (41)	60/**78**	**83/**101/119	
Acetaldehyde (44)	63/**81/**99	**89/**107/125	

* note some overlap between the ions for ROOOOR' at *m/z* **127**/145 and for ROOR at *m/z* **109**/127.

Table S3. List of the reactions and rate coefficients used in the kinetic analysis.

Model I	Model II
$CH_3O_2 + CH_3O_2 \rightarrow CH_3OOOOCH_3$ (1)<br>$CH_3OOOOCH_3 \rightarrow CH_3O_2 + CH_3O_2$ (-1)<br>$CH_3OOOOCH_3 \rightarrow CH_3O{+}CH_3O + O_2$ (2a)<br>$CH_3OOOOCH_3 \rightarrow HCHO{+}CH3OH + O_2$ (2b)<br>$CH_3OOOOCH_3 \rightarrow CH_3OOCH_3 + O2$ (2c)<br>$CH_3O + O_2 \rightarrow HCHO + HO_2$ (3)<br>$CH_3O_2 + HO_2 \rightarrow CH_3OOH + O_2$ (4)<br>$HO_2 + HO_2 \rightarrow H_2O2 + O_2$ (5)	$CH_3O_2 + CH_3O_2 \rightarrow CH_3OH + HCHO + O_2$ (6a)<br>$CH_3O_2 + CH_3O_2 \rightarrow 2\ CH_3O + O_2$ (6b)<br>$CH_3O_2 + CH_3O_2 \rightarrow CH_3OOCH_3 + O_2$ (6c)<br>$CH_3O + O_2 \rightarrow HCHO + HO_2$ (3)<br>$CH_3O_2 + HO_2 \rightarrow CH_3OOH + O_2$ (4)<br>$HO_2 + HO_2 \rightarrow H_2O2 + O_2$ (5)
k_1 = adjusted<br>k_{-1} = adjusted<br>k_2 = adjusted<br>$k_{2a} = 0.33 \times k_2$<br>$k_{2b} = 0.65 \times k_2$<br>$k_{2c} = 0.01 \times k_2$<br>$k_3 = 2 \times 10^{-15}\ s^{-1}\ cm^{-3}$ (33)<br>$k_4 = 5.2 \times 10^{-12}\ s^{-1}\ cm^{-3}$ (33)<br>$k_5 = 1.6 \times 10^{-12}\ s^{-1}\ cm^{-3}$ (33)	$k_6 = 3.5 \times 10^{-13}\ s^{-1}\ cm^{-3}$ (33)<br>$k_{6a} = 0.65 \times k_6$<br>$k_{6b} = 0.33 \times k_6$<br>$k_{6c} = 0.01 \times k_6$

Table S4. Molecular properties for $CH_3OOOOCH_3$ and CH_3O_2.

These properties were calculated with the ωB97X-D3 functional and aug-cc-pVTZ basis set in ORCA.

	$CH_3OOOOCH_3$	CH_3O_2	$CH_3OOOOCH_3$*
Vibrational frequencies (cm^{-1})	3162.72	3186.34	3162.72
	3160.06	3170.13	3160.06
	3144.53	3074.89	3144.53
	3132.68	1491.41	3132.68
	3058.12	1480.71	3058.12
	3051.89	1455.85	3051.89
	1512.79	1281.69	1512.79
	1511.53	1196.95	1511.53
	1480.82	1140.43	1480.82
	1478.79	963.03	1478.79
	1455.67	508.84	1455.67
	1454.83	131.83	1454.83
	1225.31		1225.31
	1220.37		1220.37
	1181.78		1181.78
	1178.75		1178.75
	1060.71		1060.71
	1049.39		1049.39
	948.74		948.74
	897.78		897.78
	759.86		759.86
	639.15		639.15
	575.07		575.07
	446.42		446.42
	369.11		369.11
	223.49		Internal rotation
	210.57		Internal rotation
	149.91		Internal rotation
	92.11		Internal rotation
	48.7		Internal rotation
Rotational constants (cm^{-1})	0.2466627	1.786551	0.2466627
	0.076741	0.383675	0.076741
	0.067351	0.336184	0.067351
Q (300K) cm^{-3}	1.98E+34	5.60E+31	1.57E+38

*All vibrational frequencies were reduced by an anharmonic scale factor of 0.9612 in line with ref. (*37*) and the 5 lowest ones were replaced by internal rotors. This gives a total molecular partition function of 1.57E+38 cm^{-3}.

Table S5. Calculation of the relative electronic energies (ΔE_0) between 2 CH_3O_2 and $CH_3OOOOCH_3$ and bond enthalpy of the central O-O bond in $CH_3OOOOCH_3$.

Method	ΔE_o (kJ mol^{-1})	ΔH (kJ mol^{-1})	ΔE_o (kJ mol^{-1})*
ωB97W-D3/ aug-cc-pVTZ	31.52	24.42	34.52
M06–2X / aug-cc-pVTZ	49.42	41.75	53.59
CCSD(T)/ aug-cc-pVDZ//ωB97W-D3/ aug-cc-pVTZ	50.31	43.26	-
CCSD(T)/ aug-cc-pVTZ//ωB97W-D3/ aug-cc-pVTZ	60.25	53.16	60.92
CCSD(T)/ aug-cc-pVQZ//ωB97W-D3/ aug-cc-pVTZ	59.46	52.36	61.25
CCSD(T)/ CBS/2/3)//ωB97W-D3/ aug-cc-pVTZ	65.54	58.44	68.57
CCSD(T)/ CBS(3/4)//ωB97W-D3/ aug-cc-pVTZ	59.84	52.75	62.96

*data from Salo et al. (*16*)
